# Emotional Impact of COVID-19 Lockdown Among the Spanish Population

**DOI:** 10.3389/fpsyg.2020.616978

**Published:** 2020-12-17

**Authors:** Elena Gismero-González, Laura Bermejo-Toro, Virginia Cagigal, Angustias Roldán, María Jesús Martínez-Beltrán, Lucía Halty

**Affiliations:** ^1^Department of Psychology and Clinical Unit of Psychology (UNINPSI), Comillas Pontifical University, Madrid, Spain; ^2^Department of Psychology, Comillas Pontifical University, Madrid, Spain; ^3^San Juan de Dios School of Nursing and Physical Therapy, Comillas Pontifical University, Madrid, Spain

**Keywords:** COVID-19, mass quarantine, positive affect (PA), negative affect (NA), health habits, perceived social support, resilience (psychological)

## Abstract

**Background:**

The COVID-19 pandemic has resulted in some populations being confined to their homes as part of infection control measures. This situation can be hard to cope with due to separation from loved ones, prohibition of regular activities, fear of infection, loss of freedom, and so on. These negative impacts cause considerable psychological stress, and all the more so when the situation continues for an extended period, as was the case in Spain. The present study was aimed at investigating the effects of COVID-19 quarantine on the emotional functioning of confined Spanish individuals after 8 weeks of lockdown by means of a cross-sectional study. The possible associations between changes in emotional functioning and demographic variables (age and sex), health habits (physical exercise, following a routine, and smoking), social support, and resilience were also analyzed.

**Methods:**

A total of 906 Spanish adults completed an online survey to gather information about their prevailing mood and affects (before and after 8 weeks of lockdown), using the Positive and Negative Affect Schedule (PANAS) (Watson et al., 1988), and other variables related to their habits and protective factors.

**Findings:**

As expected, the data indicated an increase in negative affects (e.g., “upset,” “afraid,” “distressed”) and a decrease in positive affects after 8 weeks under lockdown, as well as a general decline in overall mood. The largest increases in negative affects were observed in young adults (18–35 years) and women. We did not find any differences between people who were or were not diagnosed with COVID-19. Adhering to a routine, maintaining the same weight, and moderate physical exercise were associated with fewer negative affects, which indicates they are important protective factors, as are perceived social support and resilience.

**Conclusion:**

In order to mitigate the psychological impact of confinement, it is important to develop psychoeducational measures that encourage subjects to adhere to health habits and promote social support and resilience as protective factors. A special preventive focus should be placed on the most vulnerable population groups, namely women and young adults. For a public health lockdown to succeed, its negative consequences must be minimized insofar as possible through adequate knowledge of the risk factors and protective factors, and by means of prevention-oriented organization.

## Introduction

Given the epidemiological situation caused by the coronavirus disease (COVID-19) pandemic, the Spanish Government enacted a nationwide “state of alarm” (España. Ministerio de la Presidencia, Relaciones con las Cortes y Memoria Democrática, 2020) on March 14, 2020, initially for 15 days (during which total confinement was required), but successively extended until June 7. This involved the confinement of everyone except those deemed essential workers (people working in healthcare, law enforcement, transport, distribution, and sale of vital goods); therefore, most of the population could only leave their homes to buy basic necessities (groceries, medications) or due to force majeure.

Quarantine entails a difficult situation to endure, involving separation from loved ones, loss of liberties, insecurity about possibly getting infected, among others, and of course boredom, which can also have negative effects. The implementation of a mass quarantine is perceived as a sign that the situation is very serious and could get even worse. It is also associated with a perceived loss of control and the feeling of being trapped, which may be further exacerbated if family members are separated throughout lockdown (Brooks et al., 2020).

Furthermore, the overall impact of an epidemic on mental health is also associated with its degree of spread and mortality rate (Laird et al., 2019), both of which reached very significant levels in the case of COVID-19 in Spain. The Spanish public suffered lifestyle restrictions because of a lack of awareness and anticipation of the danger during the epidemic’s exponential spread (Rudan, 2020). Faced with such virulence, isolation becomes the only means of cutting transmission (Ding et al., 2020).

The literature supports that enforced isolation has a significant impact on many aspects of people’s lives, generating substantial psychological stress which, in some instances, can even trigger a range of psychological problems (Brooks et al., 2020; Castelli et al., 2020; Mazza et al., 2020). Moreover, social isolation increases the risk of morbidity and mortality, while social support contributes to mental health (Pantell and Shields-Zeeman, 2020). Out of the different psychological effects reported during past quarantine situations, the most prevalent ones were low mood and irritability (Lee et al., 2005). Confinement, the loss of contact with others, and the inability to follow normal routines produce boredom, frustration, and a sense of isolation from the rest of the world, which in turn generate anxiety and restlessness in people under lockdown (Blendon et al., 2004; Hawryluck et al., 2004; Robertson et al., 2004; Cava et al., 2005; Reynolds et al., 2008; Braunack-Mayer et al., 2013; Wilken et al., 2017). Various stressors aggravate these effects, such as the duration of the quarantine, fear of infection, frustration, boredom, inadequate information, a lack of basic provisions, economic losses, and stigma (Brooks et al., 2020).

Recent studies have determined a significant reduction in the physical activity levels of adults (Giustino et al., 2020; López-Bueno et al., 2020a), and children and adolescents (López-Bueno et al., 2020c), during the Covid-19 confinement. Moreover, such studies have found that adults who experienced higher reductions of physical activity levels, or who performed lower levels of physical activity during the Covid-19 pandemic, have poor mental health and low levels of well-being (López-Bueno et al., 2020b).

On the other hand, resilience is considered a protector of mental health and, as such, can mitigate the adverse effects of traumatic situations like a pandemic and any associated isolation (Cai et al., 2020; Conversano et al., 2020). Resilient individuals have a greater capacity to overcome adversity and recover more quickly (Horesh and Brown, 2020).

In a setting as difficult as the one in Spain during the COVID-19 pandemic, it is important to assess the general population’s mood after 2 months under lockdown. To this end, we created an online, self-administered survey that, among other variables, incorporated an instrument recognized as an excellent indicator of mood—the Positive and Negative Affect Schedule (Watson et al., 1988)—one of the most used and recommended emotion assessment tools in the literature (Thompson, 2007). It is a short, easy-to-administer instrument that overcomes the reliability and validity problems associated with previous affect assessment scales (Watson et al., 1988). The PANAS scale has shown significant correlations with depression and anxiety, positive correlations with the negative affect subscale, and negative correlations with the positive affect subscale (Watson et al., 1988; Robles and Páez, 2003; Terracciano et al., 2003; Lopez-Gomez et al., 2015). Authors have also shown that it is a good indicator of depression (Terracciano et al., 2003; Crawford and Henry, 2004). Positive affect (PA) basically corresponds to how enthusiastic, active, and alert a person feels. A high PA score reflects energy, concentration, and happiness, while a low PA implies sadness and lethargy. Negative affect (NA), however, is a general dimension of subjective stress and displeasure involving various aversive mood states such as anger, contempt, disgust, guilt, fear, or nervousness. A low NA score indicates a state of calm and tranquility (Watson et al., 1988).

This study has two main aims: to shed light on how a state of quarantine influences the emotional functioning of the confined population, as well as their emotions and mood after 8 weeks of lockdown; and to determine the consequent effects, if any, of certain demographic variables (age, sex), health habits (physical exercise, following a routine, smoking), and protective factors (social support, resilience). Learning more about these factors is important for the development of tools that will help public health systems plan measures for managing any future crises that might require similar actions, including possible resurgences of the current pandemic after lifting the lockdown.

## Materials and Methods

### Selection and Description of Participants

The study included 906 Spanish subjects. The inclusion criteria of the participants were being over 18 years of age, having an adequate understanding of the Spanish language, and being a resident of Spain. The sample comprised 72.5% women and 27.5% men (similar to the participant ratio observed by Mazza et al. (2020), which included 71.7% women and 28.3% men). Most participants (72.8%) lived in the Community of Madrid, one of the regions with the highest COVID-19 infection rates in Spain. The mean age of subjects was 43.43 years (*SD*: 13.67).

We employed accidental, non-probability, snowball sampling.

### Measures

#### Demographic Variables

Participant age, sex, and geographical area of residence.

#### Mood Variables

Positive and Negative Affect. We used the Spanish adaption of the Positive and Negative Affect Schedule (PANAS) (Watson et al., 1988) published by Lopez-Gomez et al. (2015). The PANAS tool includes two 10item subscales that measure the positive affect (PA) and negative affect (NA) through questions about affects such as “stressed,” “nervous,” or “satisfied.” Respondents used a Likert scale (from 1 = “not at all” or “very slightly” to 5 = “extremely”) to indicate if they had felt each affect during a specified period. The Cronbach’s α reliability indices for the scale ranged between 0.83 and 0.92 (Lopez-Gomez et al., 2015). In our study, we applied the PANAS tool twice, firstly to measure how respondents felt before the COVID-19 health crisis and then to determine their affects during lockdown. Our sample also presented high Cronbach’s alpha reliability coefficients: PA 0.90 and 0.91; and NA 0.86 and 0.88, for before and during lockdown, respectively.

Overall Mood. Participants were asked three questions regarding, “How would you rate your overall mood before COVID-19/during lockdown/when restrictions started to be lifted” (from 1 = “very bad” to 6 = “very good”).

#### Physical Health and Health Habit Variables

COVID-19 Positive or Negative. This was based on an ad hoc questionnaire that asked participants if they had been diagnosed with COVID-19 by means of a test (“yes” or “no”). COVID-19 positive participants were then asked about the *severity of their symptoms* (mild symptoms treated at home/moderate symptoms requiring hospitalization/severe symptoms treated in ICU) and the number of *days of recovery* (at home/hospital ward/ICU).

##### Smoking. Respondents were asked if they smoked.

Physical Activity. Evaluated using the *International Physical Activity Questionnaire* (IPAQ) (Craig et al., 2003). We used the 9-item short form of the IPAQ (IPAQ-SF) and obtained a continuous score from the questionnaire by recording exercise involving walking (3.3 METs), moderate (4.0 METs), and vigorous (8.0 METs) activity, all in MET–minutes/week, and the minutes of sedentary behavior per day. We calculated the test–retest reliability of the IPAQ-SF and found that 75% of the correlation coefficients were greater than 0.65 (Craig et al., 2003).

Weight Change During Lockdown. Participants were asked whether they had gained/lost/maintained their weight during the COVID-19 lockdown.

Time Management and Routines. Assessed using a single question, “How do you think you have managed your time (routines, etc.) during the lockdown?” (from 1 = “very badly” to 6 = “very well”).

#### Protective Factor Variables

Resilience. We used the Spanish version (Rodríguez-Rey et al., 2016) of the Brief Resilience Scale (BRS) (Smith et al., 2008). The BRS is a 6-item scale with responses given from 1 (“totally disagree”) to 5 (“totally agree”) according to a Likert-type scale. The Cronbach’s alpha coefficient for the adaptation was 0.83, while the value for our sample was 0.87.

Perceived Social Support. This variable was assessed through the question, “How would you score the social support you have received during lockdown (emotional or practical support from relatives, friends, workmates)?” (from 1 = “no support” to 5 = “a lot”).

### Procedure

We followed a cross-sectional study design, developed an online survey, and contacted subjects via email and through social media. The survey was uploaded and shared on the Google online survey platform, and sent out on May 8, 2020, when the lockdown enacted by the Spanish government due to the COVID-19 health crisis had been in force for 8 weeks. Data were collated between May 8 and May 13.

Expedited ethics approval was obtained from the Comillas Pontifical University Ethics Committee in accordance with the principles embodied in the Declaration of Helsinki. Participation in the study was voluntary and subject to the participants’ informed consent. They explicitly expressed that they agreed to participate by checking a box after reading the consent form, which explained the aims of the study, data processing, and data anonymity. Participants were told that if they so desired, they could leave the study at any time and without any negative consequences. They were also given the contact details of one of the researchers if they wanted to discuss any study-related questions.

### Data Analysis

Data were analyzed using SPSS statistical software, version 26.0. The items of the variables analyzed in this study yield data of skewness ≤ 2 and kurtosis ≤ 7, which allows to consider the normal distribution (Curran et al., 1996); furthermore, the large size of the sample allows to assume the normality of the data directly (Lumley et al., 2002). Student’s *t*-tests were performed to compare the means of the different PANAS moods, positive and negative affect, and physical activity before and during confinement. ANOVA tests were conducted to compare the means of positive and negative affect as a function of age and changes in weight. Pearson correlation coefficients were performed to study the relationship between the variables of positive and negative affect, time management, perceived social support, physical activity, and resilience. Finally, Chi-squared tests were performed to analyze the relationship between positive and negative COVID variables with health variables.

## Results

### Descriptive Statistics and Significant Differences in Mood Variables Before and During Lockdown

We performed a two-way repeated measures ANOVA and found statistically significant differences in participant-reported mood, which was lower during lockdown (Mean = 3.63; *SD* = 1.24) compared to both before the quarantine (Mean = 4.64; *SD* = 1.08) and when restrictions started to be lifted (M = 3.78; *SD* = 1.21) [*F*_(2,905)_ = 338.70, *p* = 0.0001, *d*^2^ = 0.27].

With respect to the affects, which were evaluated using the PANAS PA and NA subscales, we also observed significant differences in Student’s *t*-test. The subjects had significantly lower PA scores, both in terms of each specific affect and the total score, during lockdown than in the preceding period. Similarly, their responses during lockdown gave a higher total score on the NA subscale and statistically significant differences in all individual negative affects, apart from in the items “guilty” and “ashamed,” the scores for which did not change significantly (Table 1).

**TABLE 1:** Descriptive statistics, mean difference and effect size of positive and negative affects (PANAS) before and during lockdown due to COVID-19.

**Positive Affect (PA)**	***N***	**Min**	**Max**	**Mean before**	**Mean lockdown**	***SD before***	***SD lockdown***	**Student’s *t*-test**	***P***	***D Cohen***
PANAS 1. interest	906	1	5	3.94	3.42	0.80	1.04	13.616	0.000	0.56
PANAS 3. Excited	906	1	5	3.47	2.55	0.92	1.09	23.011	0.000	0.91
PANAS 5. Strong	906	1	5	3.49	3.09	0.92	1.02	10.834	0.000	0.41
PANAS 9. enthusiastic	906	1	5	3.22	2.36	1.04	1.08	20.983	0.000	.81
PANAS 10. Prood	906	1	5	3.51	3.21	0.94	1.09	8.668	0.000	0.30
PANAS 12. alert	906	1	5	3.66	3.25	0.85	0.97	12.130	0.000	0.45
PANAS 14. inspired	906	1	5	3.10	2.75	1.00	1.16	9.438	0.000	0.32
PANAS 16. determined	906	1	5	3.41	2.90	0.93	1.10	13.507	0.000	0.50
PANAS 17. attentive	906	1	5	3.40	2.90	0.92	1.08	13.123	0.000	0.50
PANAS 19. active	906	1	5	3.80	3.16	0.93	1.09	15.941	0.000	0.63
Total PA	906	10	50	35.00	33.16	6.71	6.34	20.574	0.000	0.28
**Negative Affect (NA)**										
PANAS 2. distressed	906	1	5	1.88	2.60	0.98	1.14	–17.394	0.000	−0.67
PANAS 4. upset	906	1	5	1.80	2.89	0.93	1.11	–25.786	0.000	−1.06
PANAS 6. guilty	906	1	5	1.39	1.40	0.76	0.82	–0.381	0.703	–
PANAS 7. scared	906	1	5	1.54	2.47	0.80	1.14	–24.057	0.000	−0.94
PANAS 8. hostile	906	1	5	1.33	1.74	0.65	1.01	–13.354	0.000	−0.45
PANAS 11. irritable	906	1	5	1.88	2.35	0.90	1.17	–12.053	0.000	−0.45
PANAS 13. ashamed	906	1	5	1.29	1.26	0.67	0.64	1.505	0.133	–
PANAS 15. nervous	906	1	5	2.14	2.54	1.05	1.23	–9.941	0.000	−0.35
PANAS 18. jittery	906	1	5	1.96	2.33	1.02	1.16	–9.733	0.000	−0.34
PANAS 20. afraid	906	1	5	1.60	2.21	0.89	1.18	–16.695	0.000	−0.58
Total NA	906	10	45	16.81	21.78	5.77	7.50	–22.014	0.000	−0.74

*PA, Positive Affect; NA, Negative Affect.*

### Descriptive Statistics for Diagnosis of COVID-19

Seventy-eight participants (8.6%) reported they had contracted COVID-19. Most presented mild or moderate symptoms (91.1%); 70 were indicated home recovery, with a mean of 14.27 days of recovery (*SD* = 11.35), seven had to be admitted to hospital, for a mean of 10.29 days (*SD* = 3.10), and one participant did not answer this question. None of the participants reported having been admitted to an ICU.

### Health Habit and Protective Factor Variables

Most participants were non-smokers (82.5%) and 45.6% reported weight changes during lockdown. Additionally, more than 70% of subjects considered that they received sufficient or significant social support (M = 3.95; *SD* = 0.901) and 68% considered that they had practiced good time management and followed a routine during lockdown. The mean value for the resilience variable was 19.88 (*SD* = 4.44), wherein the highest score was 30 and the lowest was 6.

As for physical activity, levels of intense activity, walking, and total physical activity decreased significantly during lockdown, whereas there were no significant differences in moderate activity levels. Time spent sitting increased while under lockdown (Table 2).

**TABLE 2:** Frequency of physical activity before and during lockdown.

	***N***	**Min.**	**Max.**	**Mean**	***SD***	**Student’s *t*-test**	***P***	***D* Cohen**
METs-min/week intense before	906	0	16,800	1266.53	1783.78	2.011	0.045	0.07
METs-min/week intense during	906	0	16,800	1147.68	1661.44			
METs-min/week moderate before	906	0	8,400	551.13	862.61	–0.111	0.911	–
METs-min/week moderate during	906	0	8,400	554.92	932.46			
METs-min/week walking before	906	0	6,930	1024.68	1317.48	9.509	0.000	0.34
METs-min/week walking during	906	0	6,930	613.20	1061.32			
Total physical activity before	906	0	32,130	2842.34	2924.73	5.601	0.000	0.19
Total physical activity during	906	0	32,130	2315.80	2665.32			
Time spent sitting before	906	10	300	239.99	84.83	–11.014	0.000	0.33
Time spent sitting during	906	10	300	264.76	66.432			

### Differences in Means Between COVID-19 Positive and Negative Participants Based on Mood and Health Variables

A random sample was selected from among the COVID-negative participants that was similar in size to the COVID-positive sample (*N* = 80), considering that the number of COVID-positive individuals (*N* = 78) was lower than those who had not contracted the disease.

There were no significant differences based on sex [χ^2^_(1)_ = 0.224; *p* = 0.131] or mean age with respect to whether or not participants contracted COVID-19 (*t* = 0.770; *p* = 0.442).

Similarly, we did not find any significant differences between COVID-19 positive and negative participants as regards to smoking [χ^2^_(2)_ = 2.204; *p* = 0.332].

Nor did we observe any differences between these two groups for total physical activity before (*t* = 0.194; *p* = 0.846) and during (*t* = 0.527; *p* = 0.599) lockdown. Lastly, we did not find any differences between COVID-19 positive and negative participants in PA before (*t* = 0.169; *p* = 0.866) or after 8 weeks of lockdown (*t* = 0.316; *p* = 0.752), in NA before (*t* = 0.215; *p* = 0.830) or during (*t* = −1.022; *p* = 0.308), or in perceived social support [χ^2^_(4)_ = 9.257; *p* = 0.055].

### Differences in Positive (PA) and Negative Affect (NA) During the Health Crisis Based on Demographic and Health Habit Variables

Table 3 shows that the mean PA was significantly higher among non-smokers than smokers. There were also differences in PA in association with participant weight changes, where the mean PA was higher for subjects who reported no weight change compared to those who gained weight (Table 4).

**TABLE 3:** Student’s *t*-test in positive affect (PA) and negative affect (NA) during lockdown depending on gender and smoking habit.

		***N***	**Mean**	***SD***	**Student’s *t*-test**	***p***	***D* Cohen**
**Gender**							
PA	Male	249	32.80	6.43	1.057	0.291	–
	Female	657	33.30	6.31			
NA	Male	249	19.81	7.05	–4.956	0.000	–0.37
	Female	657	22.53	7.52			
**Smoker**							
PA	No	747	33.46	6.26	3.051	0.002	0.26
	Yes	159	31.77	6.56			
NA	No	747	21.28	7.24	–4.143	0.000	–0.38
	Yes	159	24.18	8.17			

*PA, Positive Affect; NA, Negative Affect.*

**TABLE 4:** ANOVA in mean differences in positive affect (PA) an negative affect (NA) during lockdown in function of age and weight changes.

		***N***	**Mean**	***SD***	***F* (df)**	***P***
**Age**						
PA	<35	248	32.46	6.465	2.373 (2)	0.094
	36–60	586	33.49	6.358		
	>60	72	32.88	5.637		
NA	<35	248	22.67	7.400	6.013 (2)	0.003
	36–60	586	21.73	7.435		
	>60	72	19.22	7.715		
**Weight change**						
PA	None	493	33.93	5.988	9.758 (2)	0.000
	Lost	145	33.03	6.971		
	Gained	268	31.82	6.421		
NA	None	493	20.86	7.283	8.308 (2)	0.000
	Lost	145	22.84	7.808		
	Gained	268	22.91	7.486		

*PA, Positive Affect; NA, Negative Affect.*

With regard to NA, women presented a significantly higher mean than men, as did smokers in comparison with non-smokers (Table 3). We also found that age implied significant differences in NA with subjects >60 years reporting lower NA scores than the other two age groups, < 36 years and 36–60 years. Furthermore, the NA score was lower among respondents who recorded no weight change than those who reported losing or gaining weight (Table 4).

### Correlations Between PA, NA, Routines, Physical Activity, Perceived Social Support, and Resilience

There was a positive, moderate, and significant correlation between PA and time management, perceived social support, and resilience. NA, on the other hand, correlated negatively with the same variables. There were hardly any significant correlations between PA or NA and the variables associated with physical activity during lockdown (Table 5).

**TABLE 5:** Correlations between positive affect (PA) and negative affect (NA) during lockdown and time management, perceived social support, physical activity, and resilience.

	**PA**	**NA**
Time management and routines	0.392**	−0.361**
Perceived social support	0.289**	−0.161**
METs-min/week intense	0.075*	–0.026
METs-min/week moderate	0.074*	−0.079*
METs-min/week walking	0.025	–0.021
Total physical activity	0.083*	–0.052
Time spent sitting	–0.045	0.034
Resilience	0.420**	−0.407*

PA, Positive Affect; NA, Negative Affect. *Correlation is significant at the 0.05 level (bilateral). **Correlation is significant at the 0.01 level (bilateral).

## Discussion

The purpose of this study was to discover the impacts of an 8-week quarantine on the emotional functioning of the Spanish population, where total confinement was decreed for the entire country. Considering it was a long period of time, Hawryluck et al. (2004) reported that confinement for more than 10 days has important negative psychological effects compared to shorter lockdowns, as well as the consensus that lockdowns may have a broad range of substantial and long-lasting psychological effects (Brooks et al., 2020). We believe it is important to discover the extent to which demographic variables (age and sex), health habits (such as physical exercise, establishing routines, and smoking), and protective factors (perceived social support and resilience) may affect the Spanish population psychologically during lockdown.

With respect to the positive and negative affects before and during lockdown, the results of the present study show that participants’ PA scores fell and their NA scores increased while under quarantine, wherein the differences were significant and of moderate to high magnitude. Several studies have found that the greatest impact of an epidemic on mental health is the increase in anxiety, panic, anger, disappointment, sleep problems, disturbances in circadian rhythms, as well as depressive and post-traumatic stress symptoms (Halperin, 2014; Hackett and Steptoe, 2017; Aaby et al., 2020; Zhang and Ma, 2020). Specifically, confinement increases an individual’s levels of confusion, irritation, fear, pain, numbness, and anxiety-induced insomnia (Brooks et al., 2020).

Upon examining NA with respect to age, our results show that after 8 weeks of lockdown young adults (18–35) had considerably higher NA scores than middle-aged (36–60) and elderly adults (≥61), plus the middle-aged group also reported higher NA values than the elderly. This means that the age cohort with the lowest NA score after 8 weeks of quarantine were those aged over 61 years. Although over a decade earlier, Hawryluck et al. (2004) did not find any relationship between age and distress during lockdown enforced due to the SARS epidemic, and more recent data from Spain (Balluerka et al., 2020; Ozamiz-Etxebarria et al., 2020) and other countries (Huang and Zhao, 2020; Wang H. et al., 2020) also point in the same direction. This indicates the importance of interventions designed for young adults to help them with their emotional management of possible quarantines, thus preventing potential mental health complications.

As for gender, the results of our study agree with most investigations conducted in other countries, which have found that women have higher NA scores than men during lockdown, particularly anxiety, stress, and depression (Balluerka et al., 2020; Mazza et al., 2020; Oliver et al., 2020; Wang C. et al., 2020). However, other studies did not observe any association between emotional distress and sex (Huang and Zhao, 2020; Zhang and Ma, 2020), which implies, on one hand, that this point warrants further examination and, on the other, the need to pay special attention to a preventive approach to women’s mental health in the event of future lockdowns.

Lockdown resulted in a significant worsening of all the positive affects. Of note were the declines in “excited” and “enthusiastic,” although this was expected given the reduction in leisure or stimulating activities while under confinement. Regarding the negative affects, all except “guilty” and “ashamed” intensified during lockdown. The differences in “upset,” “afraid,” and “distressed” are especially relevant as they are consistent with an uncertain and threatening situation, such as the global health crisis caused by COVID-19. What is more, these differences are comparable with those published by other researchers (Mazza et al., 2020; Zhang and Ma, 2020). Balluerka et al. (2020) examined a Spanish sample and found that participants reported: (i) a very high level of uncertainty and were afraid of losing a loved one or developing a serious illness; (ii) increased levels of worry, irritability, hostility, and general distress; and (iii) mood swings, symptoms of depression, feeling unreal, trouble concentrating, and less optimism.

The present study did not find any significant differences in PA and NA scores between participants who did and did not report contracting COVID-19. This could be because none of the participants diagnosed with COVID-19 developed severe symptoms and 91% of them recovered at home without requiring hospitalization.

Very few studies have examined the relationship between mood and health habits in a lockdown situation. In our study we aimed to further our knowledge of the relationships between smoking, adhering to routines, and physical exercise (walking, moderate, and intense activity) and participant-reported PA and NA levels, in order to determine whether changes in these health habits resulted in changes in the affects felt by the subjects.

Balluerka et al. (2020) found that 24.0% of smokers reportedly smoked more, while 11.3% said they smoked less tobacco during lockdown. According to our results, smoking is related to both PA and NA, as smokers reported having lower PA levels and higher NA levels.

Weight is another factor that may be affected by changes in habits during lockdown. Lockdowns can alter physical activity and eating behaviors in such a way that they compromise human health (Ammar et al., 2020). Just over half (54.4%) of the participants in our study maintained the same weight, 26.6% gained weight, and 16.0% lost weight (which differs somewhat from the data published by Zachary et al. (2020), in which 22% gained weight, 19% lost weight, and 59% remained practically the same weight). The group that gained weight after 8 weeks lockdown reported lower PA scores than the group with the same weight, and this latter group had lower NA levels compared to the subjects who reported weight change. Eating more is a way of dealing with stress, anxiety, or boredom (Fallon, 2020; Zachary et al., 2020). The main risk factors for weight gain during quarantine are inappropriate hours of sleep, snacking after dinner, deviation from the normal diet, stress-related eating, and less physical activity (Zachary et al., 2020). The results of the present study show that people who did intense physical activity during confinement either maintained or lost weight, subjects who completed moderate physical activity did not present any weight change, and those who did less moderate or intense physical activity gained weight. We did not, however, observe any relationship between time spent sitting down and weight gain or loss; although this finding is surprising, it clearly agrees with the work of Zachary et al. (2020) who reported similar findings in an adult sample.

Here we observed that intense physical activity correlates with a higher PA score and moderate physical activity is associated with a lower NA score. Our data are coherent with those of Fallon (2020), who indicated that physical exercise is moderately more effective in reducing the symptoms of depression compared to an absence of any therapy at all and that intense physical exercise helps reduce anxiety. Another interesting result of the present study was that people with better adherence to routines completed higher levels of intense and moderate physical activity during lockdown, as well as a greater overall level of activity.

Routines, for their part, are known to contribute to emotion regulation and mental health (Fallon, 2020). In a similar vein and according to our present results, participants with stable habits have a greater PA and lower NA levels, which suggests that adhering to routines acts as a protective factor for mental health under a quarantine situation.

Regarding protective factors studies have shown the clear relationship between personal well-being and social support (Zhang and Ma, 2020). In the specific setting of a quarantine, the data from this study show that social support correlates with higher PA and lower NA scores, as well as better organization of routines. This highlights the importance of adequate social support to cope with the long days of being under lockdown. Fostering resilience is one of the best means of preventing stress and psychological distress in trying circumstances (Laird et al., 2019). Our study shows that people with greater resilience have higher PA and lower NA levels, which indicates that resilience can function as a protective factor during confinement.

Ultimately, the results show that long quarantine situations entail a risk of negative affects increasing and positive affects abating in intensity. All this can lead to the conclusion that periods of confinement imply a grave risk of psychological harm for the population. Our results, however, offer important clues about the expediency of promoting health habits (e.g., adhering to routines, exercising, maintaining the same weight) and protective factors (namely social support and resilience) among subjects, measures that would significantly help mitigate the impact of confinement. It is important to understand that a certain increase in distress levels when coping with a difficult situation, such as quarantine, is a normal adaptive response to the circumstances (Harder et al., 2020) and that, with time, they will return to pre-confinement levels.

This study has the following limitations. Firstly, the small sample size of COVID-19 positive participants, which, while being proportionally representative of the total sample, did not enable us to study certain points that seemed relevant when analyzing the impact of the lockdown because it was mainly composed of subjects who only required home rest to recover. In addition, the convenience sampling method used to recruit participants might lead to selection bias which, in turn, could have led to a biased estimation of the study variables concerning the study population. Therefore, the results of this study should be interpreted based on this information. It was not possible to use time-consuming methods to ensure a representative sample under the circumstances present at the moment of the study, and because of the need to collect data rapidly. Furthermore, all the variables are self-reported and there is still a chance of information bias. Finally, due to its cross-sectional design, the present study does not allow for inferring causal conclusions. It should be noted that all the data corresponding to participant mood prior to lockdown were recorded at the same time as those collected after 8 weeks of confinement, so the responses may be biased by the subjects’ own perception. However, it was simply not possible to collect data on pre-confinement conditions to allow for assessment of more causal associations, since the epidemic and subsequent confinement could not be foreseen.

In summary, this study set out to explore the emotional impact of lockdown during the COVID-19 crisis on the Spanish population. While increasingly more data are being collated on the psychological effects of imposing confinement on a population, this work aimed to shed light on the relationship between distress and the main demographic variables, health habits, and protective factors.

We agree with Huang and Zhao (2020) regarding the importance of implementing preventive mental health policies to reduce the impact of quarantines on the most vulnerable population groups, including young adults (18–30 years) and women, while facilitating access to psychological support. It is essential to promote psychoeducational measures that encourage adherence to routines and physical exercise habits, besides providing social support through natural support networks and placing an emphasis on developing mechanisms of resilience.

Lastly, as put forward by Brooks et al. (2020), these results underscore the responsibility of those who have to make decisions of such gravity, given that the success of this kind of public health measure depends on reducing its negative effects to the greatest extent possible, through adequate knowledge of the risk factors and protective factors, and by means of prevention-oriented organization. Awareness and understanding of the experiences of those living through lockdown will help improve disease infection control and minimize any negative effects suffered by affected individuals, families, and communities (Hawryluck et al., 2004).

## Data Availability Statement

The raw data supporting the conclusions of this article will be made available by the authors, without undue reservation.

## Ethics Statement

The studies involving human participants were reviewed and approved by the Comillas Pontifical University Ethics Committee. The patients/participants provided their written informed consent to participate in this study.

## Author Contributions

EG-G: conception, writing—original draft, and supervision. LB-T: methodology, writing—review, and editing. VC: interpretation, writing—review, and editing. AR and MJM-B: writing—review, and editing. LH: analysis of data. All authors contributed to the article and approved the submitted version.

## Conflict of Interest

The authors declare that the research was conducted in the absence of any commercial or financial relationships that could be construed as a potential conflict of interest.
